# Understanding Needs Satisfaction and Frustration in Young Athletes: Factor Structure and Invariance Analysis

**DOI:** 10.3390/ijerph17114046

**Published:** 2020-06-05

**Authors:** Diogo Monteiro, Luís Cid, Diogo S. Teixeira, Teresa Fonseca, Pedro Duarte-Mendes, Luís M. Silva, Filipe Rodrigues

**Affiliations:** 1 Sport Science Scholl of Rio Maior—Polytechnique Institute of Santarém (ESDRM—IPS), 2040-413 Rio Maior, Portugal; luiscid@esdrm.ipsantarem.pt (L.C.); frodrigues@esdrm.ipsantarem.pt (F.R.); 2 Research Centre in Sport, Health and Human Development (CIDESD), 5001-801 Vila Real, Portugal; 3 Faculty of Physical Education and Sport, Lusófona University (FEFD—ULHT), 1749-024 Lisbon, Portugal; diogo.teixeira@ulusofona.pt; 4 Center for the Study of Human Performance (CIPER), 1495-751 Lisbon, Portugal; 5 Polytechnic Institute of Guarda (IPG), 6300-035 Guarda, Portugal; tfonseca@ipg.pt; 6 Centro de Investigação Formação Inovação e Intervenção em Desporto (CIFI2D), 4200-450 Porto, Portugal; 7 Life Quality Research Centre (CIEQV), 2040-413 Santarém, Portugal; 8 Polytechnique Institute of Castelo-Branco (IPCB), 6000-084 Castelo Branco, Portugal; pedromendes@ipcb.pt; 9 Sport, Health & Exercise Research Unit (SHERU—IPCB), 6000-084 Castelo Branco, Portugal; 10 Kinesiolab, Piaget Institute, 2805-059 Almada, Portugal; luis.silva@almada.ipiaget.pt; 11 Department of Biomechanics, University of Nebraska—Omaha, Omaha, NE 68182, USA; 12 Neuromuscular Research Lab, Faculty of Human Kinetics, University of Lisbon, 1495-751 Lisbon, Portugal; 13 CLISSIS, Lusíada University of Lisbon, 1349-001 Lisbon, Portugal

**Keywords:** self-determination theory, needs satisfaction, needs frustration, structural equation modeling, sport

## Abstract

Sports research has been focused on the assessment of basic needs satisfaction, considering its absence as a representation of needs frustration. However, recent findings have suggested needs satisfaction and frustration as asymmetrical factors leading to differentiated outcomes. An accurate measurement of needs poses itself as a crucial aspect, facilitating coaches’ understanding of athlete’s motivational processes. This study aimed to examine the psychometric proprieties of the Basic Psychological Needs Satisfaction and Frustration Scale (BPNSFS) in a sample of Portuguese athletes. A multigroup analysis was conducted of gender, sport type, age, and years of sports practice. Additionally, needs satisfaction and needs frustration were tested as predictors of behavioral regulations examining the nomological validity of the BPNSFS. Data from 594 Portuguese athletes (38.6% female; M_age_ = 15.21; SD = 0.97) that represent two different sports (football and swimming) were analyzed. Confirmatory factor analysis and structural equation modeling procedures were followed to test the factor structure and nomological validity of the scale, respectively. Analyses indicated that the six-factor model provided an adequate fit (Comparative Fit Index = 0.947, Tucker–Lewis Index = 0.936, Standardized Root Mean Square = 0.039, Root Mean Square Error of Approximation = 0.048 (CI 90% = 0.043, 0.054)). Moreover, the multigroup analysis suggested invariance in the observed structure across groups. In addition, findings indicated a strong prediction between needs satisfaction and autonomous forms of motivation, whereas needs frustration predicted significantly controlled forms of motivation. The sport-adapted BPNSFS in a sample of Portuguese athletes seemed to be an adequate measure for the assessment of basic psychological needs satisfaction and frustration. Our findings suggested that this scale may be worth testing in future research in the sport context.

## 1. Introduction

Self-determination theory (SDT; Ryan & Deci) [1] is one of the most contemporary motivational theories used in behavioral psychology [2]. This theoretical framework is a prominent macro-theory of motivation in the field of sport psychology that describes the progression through which motivation grows, and how it impacts human behavior and well-being [1,3]. Within SDT research, basic psychological needs (BPNs) stand out as being the “heart” of motivation, in which the meeting of these needs is necessary for optimal human functioning [4]. Over the years, basic psychological needs have been extensively studied and applied in several domains [2]. It has been shown that regardless of gender, age, race, ethnicity, or religion, every individual possesses these needs since they are innate to all human beings [1]. Hence, every athlete needs this drive for fulfillment to experience adaptive outcomes when it comes to sport participation.

According to SDT, individuals are motivated by three BPNs, namely the needs for autonomy, competence, and relatedness. Autonomy represents the need to act in line with one’s own interest while feeling psychologically free; competence is defined by the need to feel a sense of mastery and the ability to attain desired outcomes; and relatedness represents the need to feel connected with others and a sense of closeness and belonging [1,2]. The level to which all needs are met has a positive effect on the sport setting. Several studies have shown that the satisfaction of all three needs is a strong predictor of positive outcomes, such as self-determined motivation [5], enjoyment [6], intentions to continue sport practice [7], and persistence [8].

In considering past literature, researchers argued that low scores on the measures of needs satisfaction did not adequately capture the intensity of mal-adaptive consequences [9]. For instance, individuals who express low needs satisfaction may display low levels of excitement and interest in certain activities. However, an individual who feels that their needs being frustrated could be related to more controlled forms of motivation, such as feeling pressured to act or feeling rejected by others. Therefore, according to Ryan and Deci [1], needs frustration can occur due to individual active engagement with the environment. Thus, a distinction between needs satisfaction and needs frustration has to be made because low levels of BPN satisfaction might not be representative of high levels of BPN frustration [2,9]. The frustration of autonomy represents a feeling of being controlled by external forces; competence frustration is defined by a sense of doubt and failure in one’s abilities; and relatedness frustration is the feeling of loneliness and social exclusion [2,10,11].

SDT proposes that motivation is dependent on the degree to which basic needs are met. Thus, how individuals will regulate their motivation towards a given behavior is explained by the satisfaction or frustration of their needs. This implicit motivation regulates behavior, and the extent of this engagement could be fully or partially explained by the quality of motivation that one holds [12]. In general, SDT explores behavioral regulations in a motivational continuum that varies from less to more self-determined forms [13,14]. Hence, the internalization and integration of the behavior could allow extrinsically motivated behaviors to become more self-determined over time [1]. In light of the above theoretical assumptions, BPN satisfaction and frustration have been theoretically and empirically connected to variations in self-determined behavior [1,2]. In the sport context, athletes who feel that their needs are being satisfied are able to engage in sport practice because of self-determined regulations, where the behavior is fully recognized in personal values and beliefs, thereby integrating the behavior into an individual’s routine [6,7,9]. Contrarily, BPN frustration has been associated with less determined forms of motivation, where the individual endorses the behavior because of external contingencies or self-imposed pressures [9].

### 1.1. Assessment of Needs

Studies aiming to adapt/validate tools for the assessment of BPN satisfaction and frustration in various contexts have increased with time [2]. Considering needs fulfillment or frustration to be an important aspect of optimal human functioning or maladaptive consequences, respectively, their assessment has also been a key theme in the sport context [15]. Nevertheless, limited research has been conducted into needs frustration, and consequentially its measurement in the sport context [10].

Based on theoretical assumptions, Chen et al. [11] developed the Basic Psychological Needs Satisfaction and Frustration Scale (BPNSFS), testing its factor structure in four different countries (i.e., Belgium, China, the USA, and Peru). Their results indicated that the effects of needs satisfaction and needs frustration were equivalent across the four countries. Since the development of the BPNSFS, research has taken a great step towards measuring needs. Several studies have been conducted to translate and validate this scale in different languages and domains, namely: (a) in the general domain, Dutch [16], German [17], Hebrew [18], Italian [19], Japanese [20], Portuguese [21], and Spanish [22,23]; in the physical education domain, Persian, Dutch, and Estonian [24,25,26]; (c) in training settings, Dutch and English [27]; (d) in the sports context [28,29]; (e) physical exercise in gym and health clubs [30]; (f) as well as in other domains, such as work [31] and space travel [32]. All of the aforementioned studies supported the factorial validity of the BPNSFS, which means that this measure is a reliable source of needs assessment and thus applicable in different cultures and contexts [2].

### 1.2. Present Study

In Portugal, Cordeiro et al. [21] were the first to translate and validate the BPNSFS in a sample of Portuguese undergraduate students. These authors found the BPNSFS to be multidimensional, explaining that this measure taps well into the assessment of needs satisfaction and needs frustration as a distinct construct over time. A limitation of Cordeiro et al.’s [21] study was the generality of this scale; that is, it was reliable for measuring needs in students, but its application in specific domains could present biased results. Additionally, no results of measurement invariance were presented, limiting its interpretation to groups with different characteristics.

Based on these limitations and Teixeira et al.’s [33] suggestions, Rodrigues et al. [30] translated the BPNSFS and validated it in a sample of physical exercisers. The results provided by Rodrigues et al. [30] showed that the original factor structure of the 24 items from the original version provided acceptable psychometric properties, as well as adequate construct validity [11]. In SDT research, Ryan [34] explains the need to use context-validated scales to measure constructs inherent to the SDT framework. In this regard, critical methodological limitations associated with the non-adaptation of questionnaires could compromise results displayed in future studies [35]. Thus, none of the previously validated Portuguese versions should be considered when assessing athletes’ needs, which means that neither Cordeiro et al. [21] nor Rodrigues et al. [30] should be used in a sports context [35,36]. 

In sport settings, the BPNSFS has only recently been applied through studies conducted by Delreu et al. [28,29]. However, if we look at the research questions and objectives proposed by these authors, none focused on the psychometric properties of the BPNSFS in the sport context. The first study performed by Delreu et al. [28] examined the extent to which the effects of autonomy-supportive and controlling coaching styles depend on the situation at hand, as well as athletes’ personal motivation in a sample of 101 judokas. The second study adopted a “helicopter perspective” towards motivating (including autonomy support and structure) and demotivating (including control and chaos) coaching across five independent samples from several individuals, team coaches, and athletes [29].

In both studies [28,29], the authors adapted the meaning of items and revised the BPNSFS to shorter versions. Specifically, Delreu et al. [28] considered an adapted six-item measure, and Delreu et al. [29] a 12-item option. Their results showed that two factors could be retained, explaining more than 40% of the variance, with items loading in their prespecified factors (i.e., needs satisfaction and needs frustration). Delreu et al. [28,29] also reported adequate internal consistency using the Cronbach’s alpha coefficient in both needs satisfaction and needs frustration factors; however, they did not fully correspond to the validation of the BPNSFS in the sport setting due to the limited use and analyses of this scale. Shorter versions were created, and no measurement invariance analyses were conducted to examine the applicability of this scale in groups with different characteristics.

Beyond the statistical assumptions, this issue must also be supported from a theoretical point of view. It is hypothesized that BPNs are said to be universal and inherent to all human beings, regardless of age, gender, cultural background, and other sociodemographic variables [1,2]. Thus, it is assumed that all human beings would perceive and experience needs satisfaction and needs frustration in an equivalent manner. However, as previous authors have mentioned, more studies are warranted on the assessment of needs across groups, to support the assumptions of universality of BPN satisfaction and BPN frustration [1]. Hence, as previously stated, SDT research must be performed within its specific context, and the questionnaires/scales used or validated in one context should not be used in another context before appropriate factor structure analysis of the measurement. Hence, Ryan [34] recommends the development and use of appropriate questionnaires for each specific context.

### 1.3. Current Study

The purpose of this study is to examine the psychometric properties of the BPNSFS in a sample of Portuguese athletes taking into consideration previous limitations. The present study considered an exercise-validated measure due to the representation of physical activity similar to sport [30]. Confirmatory factor analysis was performed to test a 24-item model, tapping into six factors, in accordance with the original measurement model of the BPNSFS [11]. Multigroup analysis was conducted to examine the applicability of this scale in groups with different characteristics, such as gender, sport type, age, and years of sports practice. 

In agreement with several authors [2,11,30], we hypothesized that: (a) the adaptation of the BPNSFS Portuguese version to the exercise domain would display an acceptable fit to all athlete samples under analysis; and (b) the multigroup analysis would show measurement invariance across groups [30].

Additionally, nomological validity analysis was conducted, assessing the relationships between BPN satisfaction and frustration and behavioral regulations based on the SDT framework (i.e., self-determined motivation vs. non-self-determined motivation) using structural equation modeling procedures. Based on previous research findings [15,33,37]), we proposed the following hypotheses: (a) BPN satisfaction would be positively correlated with self-determined motivation and negatively with less self-determined motivation; and (b) BPN frustration would be negatively associated with self-determined motivation and positively with less self-determined motivation.

## 2. Materials and Methods

### 2.1. Participants

A sample of 594 Portuguese athletes (38.6% female), from two different sports (football and swimming) and aged 10 to 17 (M = 15.21; SD = 0.97) participated in this study. Their sports experience ranged from 1 to 10 years (M = 6.02; DP = 3.15), weekly training ranged from 1 to 8 sessions (M = 3.56; DP = 1.02), and training volume varied between 60 and 120 min (M = 96.08; SD = 30.21).

To test measurement invariance of the BPNFS, groups were created based on theoretical and empirical assumptions, namely, (a) age groups were created based on the Development Model of Sport Participation [38]; (b) years of sports practice, considering the 10-year rule (i.e., <10 and ≥10 of experience) as suggested by Ericsson [39]; (c) gender (male and female); and d) type of sport (football vs. swimming). Data were collected from footballers and swimmers because they represent the two most-practiced sports in Portugal [40]. For detailed information on group characteristics, see Table 1.

### 2.2. Instruments

The Basic Psychological Needs Satisfaction and Frustration Scale Portuguese version in the exercise context was used [30]. This scale is comprised of 24-items grouped into six factors (four items each), reflecting how individuals feel how their needs are being satisfied or frustrated. Items were rated on a 5-point scale ranging from 1 (Completely Disagree) to 5 (Completely Agree).

The Behavioral Regulation Sport Questionnaire Portuguese version in the sport context was also used [41]. This 24-item questionnaire measures all behavioral regulations proposed by the motivational continuum, namely: amotivation, external regulation, introjected regulation, identified regulation and intrinsic motivation, and underlying SDT framework [1]. Items are rated on a 7-point scale from 1 (not at all true) to 7 (very true).

### 2.3. Procedures: Data Collection

Ethical approval was obtained from the ethic and scientific board of the Research Centre in Sport, Health and Human Development (reference ID: UID04045/2020) before data collection and procedures were conducted in accordance with the Declaration of Helsinki and its later amendments. Then, sport club managers were contacted and informed about the purpose of the study. After obtaining approval, coaches were contacted in order to arrange an adequate schedule for data collection. Parents and legal guardians who agreed to the participation of their children gave written informed consent. Participants were requested to complete the questionnaires before their training session in a quiet environment. Participants were told their responses to the surveys would be anonymous. The survey took nearly 15 min to complete.

### 2.4. Procedures: Adaptation of the BPNSF to the Sport Context

The adaptation of the item meaning from the exercise measure to the sport context was conducted by a panel of four experts (sports researcher, sports psychologist, researcher, and Portuguese teacher) [30]. These experts evaluated item meaning and syntax to adjust the exercise terms to the sport context without modifying their semantic content, specifically:

1. Autonomy Satisfaction: “I have a feeling of choice and freedom in the exercises I do” (exercise version); “I have a sense of choice and freedom in the way I train” (adaptation to sport);
2. Autonomy Frustration: “I feel pressured to do certain exercises” (exercise version); “I feel pressured to do certain training exercises” (adaptation to sport);
3. Relatedness Satisfaction: “I experience a warm feeling with the people I relate to in the gym” (exercise version); “I experience a warm feeling with my teammates” (adaptation to sport);
4. Relatedness Frustration: “I feel that in the gym, the people who are important to me are cold and distant with me” (exercise version); “I feel that in my club/team, the people who are important to me are cold and distant with me” (adaptation to sport);
5. Competence Satisfaction: “I feel confident that I can do the exercises properly” (exercise version); “I feel confident in the way I train and compete” (adaptation to sport);
6. Competence Frustration: “I feel insecure about my abilities during gym workout” (exercise version); “I feel insecure about my abilities during training and competition” (adaptation to sport);

### 2.5. Plan of Analysis

First, using SPSS v.23 (IBM, Armonk, NY, USA), descriptive statistics, as well as bivariate correlations, were calculated for all studied variables. Then, a Confirmatory Factor Analysis (CFA) was performed to test the factor structure of the adapted BPNSFS to the sport context. Consequently, Structural Equation Modelling (SEM) specification was performed to test the nomological validity between needs satisfaction and frustration and different types of motivation. Possible collinearity issues were diagnosed using the Variance Inflation Factor (VIF) and the tolerance test. Scores below 10 for VIF and values above 0.10 for the tolerance test ensured the appropriate conditions to test regression models [42].

Recommendations of several authors were followed for SEM analysis [42,43,44,45]. For nomological validity assessment, a two-step approach based on the recommendations of Kline (2016) [43] was followed: (1) test the factor structure of the model; (2) examine the proposed associations between the constructs under analysis. A bootstrap resampling procedure (1000 samples) was performed considering a Confidence Interval at 95% (CI 95%) to assess significance. An effect was considered significant (≤0.05) if its CI 95% did not include zero, as suggested by Hayes [46]. CFA and SEM procedures were conducted using AMOS v23 (IBM, Armonk, NY, USA).

The evaluation of measurement and structural model fitting was performed throughout the following parameters: traditional goodness-of-fit indexes including the Comparative Fit Index (CFI); Tucker–Lewis Index (TLI); Standardized Root Mean Square (SRMR); and Root Mean Square Error of Approximation (RMSEA) with its respective Confidence Interval at 90% (CI 90%). The following cutoffs were indicative of acceptable model fit: CFI and TLI ≥ 0.90 and SRMR and RMSEA ≤ 0.08 [42,43,44,45]. 

In order to examine internal consistency, composite reliability coefficients were calculated using the Raykov [47] formula adopting scores ≥ 0.70 as acceptable values. The Average Variance Extracted (AVE) was considered following the criteria of ≥0.50 as indicative of convergent validity [42]. Discriminant validity was achieved if the score did not exceed AVE values using the squared correlations between constructs [42].

### 2.6. Multi-Group Analysis

To determine whether the measurement model could be equivalent in groups with different characteristics, a multigroup analysis was performed [36,43]. Invariance procedures suggested by Byrne [43] were followed: (1) the measurement model should provide adequate fit in each sample; and, (2) configural, metric, scalar, and residual invariance criteria should be respected. A change of less than 0.01 in CFI, 0.015 in RMSEA, and 0.03 in SRMR provided evidence for metric invariance. A change of less than 0.01 in CFI, 0.015 in RMSEA, and 0.015 in SRMR provided evidence for scalar invariance as suggested by several authors [11,43,48]. 

## 3. Results

### 3.1. Preliminary Analysis

A preliminary inspection of the data showed no missing values or univariate and multivariate violations. Skewness and kurtosis values were contained between −2 to +2 and −7 to +7 respectively, which revealed no violation for univariate data distribution. However, a Bollen–Stine bootstrap with 2000 samples had to be performed since the coefficient for multivariate kurtosis exceeded the expected value (>5.0). Results showed that both the VIF test and tolerance test scores respected previously reported cutoffs ensuring the appropriate conditions to test the structural model.

### 3.2. Descriptive Statistics and Construct Validity

Data indicated higher means for needs satisfaction factors compared to needs frustration factors. All composite reliability values were satisfactory, and data indicated convergent validity in five out of six AVE values. Autonomy satisfaction did not achieve convergent validity because the score was below cutoffs. Regarding the assessment of discriminant validity, data revealed some issues involving: autonomy satisfaction—competence satisfaction; autonomy frustration—competence frustration; and, competence frustration—relatedness frustration. For detailed information, see Table 2.

### 3.3. Factor Structure Analysis

Results of the measurement model suggested an adequate fit to the data, as shown in Table 3. Specifically, the 24-item model consisting of six factors had an acceptable fit to the data in the total sample (i.e., Model 1) as well as in the samples under analysis (i.e., Models 2–9). Examination of the structural model conducted to test the nomological validity between needs satisfaction and frustration and behavioral regulations also showed an acceptable fit (i.e., SEM model).

### 3.4. Nomological Validity Analysis

The standardized coefficients displayed exciting results (see Table 4). First, BPN satisfaction predicted positively all forms of self-determined motivation as theoretically proposed. Similar patterns were found among the relationships among needs frustration and external regulation, and amotivation. Curiously, the coefficients of needs satisfaction and needs frustration on introjected regulation were not significant. Overall, nomological validity was achieved as it was previously hypothesized.

### 3.5. Multigroup Analysis

The configural model was compared to the metric model in all groups to test measurement invariance. As the results tend to show, differences (∆) between criteria were below cutoffs, moving on testing scalar invariance. Then, the configural model was compared with the scalar model, presenting changes in CFI, SRMR, and RMSEA within range (i.e., ∆ > 0.01). Lastly, the configural model was compared with the residual invariance model. While changes in CFI were above the critical cutoff in each model comparison, one should not assume that this criterion is optional as will be discussed further. Thus, analyses suggest that invariance remained stable with each subsequent model constraint (see Table 5), achieving acceptable measurement invariance between groups under analysis. 

## 4. Discussion

This study aimed to examine the psychometric properties of the BPNSFS in the sport context. We also explored whether the measurement model would provide invariance across groups with different characteristics. Additionally, nomological validity analyses were performed, analyzing the predictive power of needs satisfaction and needs frustration on all behavioral regulations proposed by the SDT framework [1]. The results generally provided adequate adaptation of the BPNSFS to the sports domain in all samples under analysis, suggesting that measurement invariance across groups was fully supported. Nomological validity was attained since the relationships across needs satisfaction and needs frustration with different types of motivation were significant overall. These findings align with previous research, which will be discussed in the following sections.

### 4.1. Factor Structure of the Sport-Adapted BPNSFS

Means revealed that athletes valued needs satisfaction more than needs frustration during their sports practice. Note that several studies in other physical activity domains such as exercise [30,33], physical education [24,26], and sport [29] have found similar results. As it stands, athletes feel that their needs are being fulfilled, and their needs are not being frustrated.

Composite reliability coefficients were above cutoffs (>0.70), displaying adequate internal consistency. Hence, these items are reliable in measuring their pre-specified factor, suggesting distinct meanings between items. Additionally, items significantly loaded their respective factor (*p* < 0.05), and factor loadings were greater than or equal to 0.50. Thus, each item explains at least 25% of latent factor variance as recommended by Hair et al. [42].

With regard to convergent validity, results showed that five out of six AVE values respected previously reported cutoffs [49]. Autonomy satisfaction AVE scores below 0.5 (AVE = 0.42) raised some validity issues. Similar results have been reported by other researchers [11,20,30], suggesting that items are not related as they are theoretically expected to be. However, items measuring autonomy satisfaction loaded their respective factor significantly (*p* < 0.05), and no cross-loadings were detected. Thus, following previous assumptions [43], items should be retained within the specified factor and moved forward to examining discriminant validity.

Some issues were found when examining the discriminant validity (i.e., autonomy satisfaction—competence satisfaction; autonomy frustration—competence frustration; and competence frustration—relatedness frustration) of the factors since the square correlation between them was higher than the AVE value in each factor [42]. Previous studies have also reported similar issues concerning these factors [30]. As a consequence, statistically speaking one would argue that these needs do not seem to be distinguishable. 

Given the potential explanatory role of autonomy, competence, and relatedness as a unifying score, cumulative research has shown that BPNs could act as a composite score when it comes to measuring motivational patterns [6,33]. As a matter of fact, according to Ryan and Deci [1], needs are co-dependent, and thus the satisfaction of only one need is not enough for optimal functioning. Current results displayed discriminant issues within the realm of needs satisfaction or needs frustration. Thus, athletes could have some issues expressing their need for autonomy satisfaction and competence satisfaction, as they arguably could be experienced as a global needs fulfillment. Similar trends could be observed for needs frustration. Results tend to show a lack of discriminant validity between all needs frustrations; thus, athletes seem to experience needs frustration as a global factor, in which the frustration of one need is related to the others. Future work is needed to examine the possible distinctiveness between each BPN in the sport context.

Overall, the 24-item, six-factor structure of the BPNSFS adapted to the sport context provided an adequate fit to the data following previous recommendations [42,43,45]. This evidence regarding the factor structure of needs satisfaction and needs frustration supports previous literature in different domains and cultures [19,20,30,50]. Hence, this scale seems to be a reliable measure in Portuguese athletes, showing that the adaptation worked well.

### 4.2. Nomological Validity

Needs satisfaction and needs frustration have been shown to yield positive and negative associations with different types of motivation, respectively. More specifically, coefficients between needs satisfactions positively predicted self-determined motivation (i.e., identified and integrated regulation and intrinsic motivation) and negatively predicted non-self-determined motivation (i.e., amotivation and external regulation). In contrast, needs frustration positively predicted less self-determined motivation and self-determined motivation. These results are in line with the SDT framework, which says that when athletes experience psychological needs fulfillment, they regulate their motivation in a more self-determined manner. In contrast, when individuals feel that their basic psychological needs are being frustrated, they tend to regulate their behavior in a less self-determined way [1,2]. Additionally, these results are consistent with previous research [10,51,52,53] showing the adaptive outcomes are contingent on needs satisfaction, and the negative consequences are related to needs frustration.

Overall, the autonomy satisfaction and frustration factors proved to be the most reliable predictors of both self-determined motivation (identified and integrated regulation and intrinsic motivation) and less self-determined motivation (external regulation and amotivation), followed by competence and relatedness. This supports the assumptions of the SDT model, in which the “founding fathers” state that autonomy is a strong predictor for developing self-determined motivation [1]. Monteiro et al. [54], in a study conducted with 1812 athletes from different sports, found similar results, showing that the need for autonomy was a stronger predictor of identified and integrated regulation, as well as intrinsic motivation, than the need for competence and relatedness. 

Interestingly, neither needs satisfaction nor needs frustration predicted significantly introjected regulation. From a conceptual point of view, one possible explanation could be related to the dual process of introjecting regulation as avoidance (i.e., more proximal to identified regulation) or approach (i.e., more proximal to external regulation) as suggested by Assor, Vansteenkiste, and Kaplan [55]. Athletes could have trouble defining their motivation of approach (“I will try to get a good performance”) or avoidance (“I will try to avoid getting negative feedback”), not knowing how their needs could predict their motivation towards sports practice. Thus, future research should carefully examine the distinctiveness of introjected regulation for avoidance and approach and analyze their association with BPN.

### 4.3. Multigroup Analysis

Assessment of the BPNSFS equivalence is essential given that substantive group comparisons are scarce and require needs satisfaction and needs frustration to have the same meaning across groups with different characteristics. Based on current results, the measurement model fits the data well in all samples under analysis. Specifically, findings provided invariance criteria for: configural invariance (i.e., the model structure was the same across groups); metric invariance (i.e., regression weights were equal across groups); and scalar invariance (i.e., item intercepts were equal across groups). Residual invariance (i.e., factor loadings, factor variances, factor covariances, and error variances are equal across groups) was the only criterion that displayed differences above cutoffs among configural and nested models. However, it is worth mentioning that several authors have stated that residual invariance criteria are optional since they are hard to achieve in the social and behavioral sciences [42,43,48]. Thus, as regards the results of the multigroup analysis across sport types, age, gender, and level of experience, the measurement model withholds equivalence [43,48,56]. 

Present evidence further supports SDT assumptions regarding the measurement of basic psychological needs as universal constructs for all human beings [1], regardless of their characteristics or cultural background. Hence, the BPNSFS seems to be a robust measure of needs satisfaction and needs frustration in groups with different characteristics, as shown in previous studies [2,11,30]. 

### 4.4. Limitations

Although the present study contributes to a better understanding of needs measurement in the sport context, current evidence should be considered in light of some limitations. The cross-sectional design limits the interpretation of causality. Thus, it would be essential to carry out longitudinal studies to further examine the effects of needs satisfaction and needs frustration in athletes during a competitive season. In the present study, only young athletes were considered, as they are more accessible to recruit than adults at the professional stages. Forthcoming studies should consider assessing other age groups and comparing differences between them to examine scale stability. Interesting findings could be exhibited, as athletes tend to evolve along with their sports practice [38], thereby possibly presenting different degrees of needs satisfaction and needs frustration. Finally, the present study was conducted with athletes from only two sports, one team (football) and one individual (swimming) sport; hence, current results cannot be generalized to other forms of sports practice. Nevertheless, the factor structure was equivalent between sports. Further research should seek to implement the BPNSFS for other types of sports or sociodemographic characteristics (e.g., economic status, ethnicity) to quantify the multidimensionality of this scale in the sport context.

## 5. Conclusions

Current evidence gives support to the Rodrigues et al. [30] scale validation in the exercise context, as well as further validation of the BPNSFS as a reliable measure of BPN satisfaction and frustration assessment. This study is of particular interest since it gives further insight into how athletes experience needs satisfaction and needs frustration as distinct constructs [9]. It is worth mentioning that this study was the first to attempt an examination of the 24-item factor structure of the BPNSFS in the sport context. Additionally, measurement invariance across groups with different characteristics was tested, something that to date has been under-researched. Overall, the current study contributes to the dissemination of the SDT-based research framework. 

Scale assessment is a complex and constant process, and should not be viewed as a strict and definite approach. This study opens doors to future instrument examination, using more advanced statistical methodologies, showing their strengths and weaknesses when defining items and targeting constructs. Thus, adaptation and validation of instruments from the original versions should be considered before conducting other analyses to avoid an uncontrolled proliferation of instruments that evaluate the same constructs’ authors [57].

Our results give sport coaches a reliable source of measurement for evaluating needs satisfaction and needs frustration in the sport domain, removing an existing gap in the literature. Coaches can confidently use the adapted and validated BPNSFS in female and male athletes, among a range of 13- to 17-year-old young individuals, with more and less than ten years of sport experience, including football or swimming practice.

## Figures and Tables

**Table 1:** Group characteristics.

Groups	N	Ages	Gender		Training Experience (Years)	Weekly Training (Sessions)	Volume Per Session (Minutes)
			male	female			
Specializing Years	317	13–15 (M = 14.65; SD = 0.479)	173	144	1–10 (M = 5.26; SD = 2.95)	2–8 (M = 3.54; SD = 1.13)	60–120 (M = 94.81; SD = 11.12)
Investment Years	277	16–17 (M = 16.14; SD = 0.345)	192	85	1–10 (M = 6.89; SD = 3.15)	3–8 (M = 3.58; SD = 0.896)	60–120 (M = 93.65; SD = 10.02)
Male	365	10–17 (M = 15.35; SD = 1.14)	365	-	1–10 (M = 7.02; SD = 3.05)	2–8 (M = 3.53; SD = 0.982)	60–120 (M = 95.61; SD = 11.22)
Female	229	10–16 (M = 14.62; SD = 2.61)	229	-	1–10 (M = 4.43; SD = 2.61)	2–8 (M = 3.60; SD = 1.10)	60–120 (M = 95.89; SD = 12.01)
Football	303	15–17 (M = 15.95; SD = 0.547)	196	107	1–10 (M = 6.60; SD = 0.321)	3–8 (M = 3.72; SD = 1.08)	60–120 (M = 97.74; SD = 10.36)
Swimming	291	10–15 (M = 14.15; SD = 1.41)	169	122	1–10 (M = 5.42; SD = 2.98)	2–8 (M = 3.40; SD = 0.950)	60–120 (M = 102.81; SD = 13.14)
<10 years of experience	405	10–17 (M = 14.81; SD = 1.45)	200	205	1–7 (M = 4.17; SD = 1.93)	1–7 (M = 4.17; SD = 1.93)	60–120 (M = 93.73; SD = 14.17)
≥10 years of experience	189	10–17 (M = 15.61; SD = 1.07)	165	24	10	3–8 (M = 3.54; SD = 0.841)	60–120 (M = 103.97; SD = 15.21)

**Note.***N* = sample size; M = mean; SD = standard deviation.

**Table 2:** Descriptive statistics, internal consistency, convergent and discriminant validity.

Constructs	M	SD	CR	FL	AVE	1	2	3	4	5	6	-
1. Autonomy Satisfaction	4.02	0.57	0.73	0.50–0.73	0.42	1	-	-	-	-	-	-
2. Competence Satisfaction	4.02	0.68	0.83	0.67–0.78	0.55	0.71	1	-	-	-	-	-
3. Relatedness Satisfaction	4.24	0.53	0.84	0.70–0.83	0.57	0.42	0.35	1	-	-	-	-
4. Autonomy Frustration	1.80	0.72	0.79	0.66–0.75	0.50	0.35	0.32	0.07	1	-	-	-
5. Competence Frustration	1.56	0.60	0.80	0.68–0.77	0.51	0.25	0.48	0.06	0.55	1	-	-
6. Relatedness Frustration	1.89	0.72	0.80	0.63–0.78	0.51	0.19	0.29	0.14	0.48	0.58	1	-
**Bivariate Correlations**												
	1	2	3	4	5	6	7	8	9	10	11	12
1. Autonomy Satisfaction	1	-	-	-	-	-	-	-	-	-	-	-
2. Competence Satisfaction	0.53 **	1	-	-	-	-	-	-	-	-	-	-
3. Relatedness Satisfaction	0.66 **	0.51 **	1	-	-	-	-	-	-	-	-	-
4. Autonomy Frustration	−0.44 **	−0.23 **	−0.46 **	1	-	-	-	-	-	-	-	-
5. Competence Frustration	−0.32 **	−0.34 **	−0.44 *	0.55 **	1	-	-	-	-	-	-	-
6. Relatedness Frustration	−0.37 **	−0.22 **	−0.57 **	0.59 **	0.61 **	1	-	-	-	-	-	-
7. Amotivation	−0.22 **	−0.19 **	−0.34 **	0.46 **	0.54 **	0.43 **	1	-	-	-	-	-
8. External Regulation	−0.16 **	−0.004	−0.26 **	0.43 **	0.34 **	0.39 **	0.48 **	1	-	-	-	-
9. Introjetced Regulation	−0.03	−0.001	−0.11 **	0.24 **	0.20 **	0.28 **	0.10 *	0.30 **	1	-	-	-
10. Identified Regulation	0.21 **	0.21 **	−0.24 **	−0.15 **	−0.17 **	−0.10 *	−0.32 **	−0.05	0.41 **	1	-	-
11. Integrated Regulation	0.42 **	0.26 **	0.42 **	−0.39 **	−0.27 **	−0.28 **	−0.37 **	−0.24	0.23 **	0.58 **	1	-
12. Intrinsic Motivation	0.43 **	0.29 **	0.49 **	−0.44 **	−0.30 **	−0.32 **	−0.41 **	−0.26	0.08 *	0.48 **	0.70 **	1

**Notes.** M = Mean; SD = Standard Deviation; CR = Composite Reliability; FL = Factor Loadings; AVE = Average Variance Extracted; the first half of table represents the square correlation across needs satisfaction and frustration; the second half of the table represents bivariate correlations across needs satisfaction and frustration and types of motivation; * *p* ≤ 0.05; ** *p* ≤ 0.01.

**Table 3:** Goodness-of-fit indexes of Basic Psychological Needs Satisfaction and Frustration Scale (BPNSFS) in all samples under analysis.

Models	χ^2^	df	χ^2^/df	*B*-*S* p	SRMR	TLI	CFI	RMSEA	CI-90%
Model 1—total sample	567.20	237	2.39	<0.001	0.039	0.936	0.947	0.048	[0.043–0.054]
Model 2—male	462.25	237	1.95	<0.001	0.045	0.937	0.946	0.051	[0.044–0.058]
Model 3—female	414.34	237	1.75	<0.001	0.052	0.900	0.912	0.057	[0.048–0.066]
Model 4—specializing years	479.70	237	2.02	<0.001	0.055	0.900	0.912	0.057	[0.050–0.064]
Model 5—investment years	407.61	237	1.72	<0.001	0.049	0.928	0.938	0.051	[0.043–0.059]
Model 6—swimming	475.89	237	2.01	<0.001	0.058	0.901	0.911	0.059	[0.051–0.067]
Model 7—football	418.78	237	1.77	<0.001	0.046	0.936	0.945	0.050	[0.042–0.058]
Model 8—≥10 years exp	458.46	237	1.93	<0.001	0.043	0.935	0.944	0.048	[0.041–0.055]
Model 9—<10 years exp	427.42	237	1.80	<0.001	0.060	0.900	0.910	0.065	[0.055–0.075]
SEM Model	2629.35	1029	2.56	<0.001	0.047	0.902	0.913	0.051	[0.049–0.054]

**Notes.** χ^2^ = chi-squared; df = degrees of freedom; χ^2^/df = normalized chi-squared; SRMR = Standardized Root Mean Square Residual; TLI = Tucker–Lewis index; CFI = Comparative Fit Index; RMSEA = Root Mean Squared Error of Approximation; CI 90% = Confidence Interval at 90%.

**Table 4:** Regression paths between needs satisfaction and frustration and types of motivation.

Path	β	CI 95%
*Needs Satisfaction*		
Autonomy → Amotivation	−0.57	[−0.710; −0.419]
Autonomy → External	−0.46	[−0.566; −0.342]
Autonomy → Introjected	0.11	[−0.021; 0.236]
Autonomy → Identified	0.91	[0.823; 0.972]
Autonomy → Integrated	0.87	[0.818; 0.912]
Autonomy → Intrinsic motivation	0.90	[0.852; 0.946]
Competence → Amotivation	−0.61	[−0.735; −0454]
Competence → External	−0.50	[−0.597; −0.390]
Competence → Introjected	−0.01	[−0.151; 0.141]
Competence → Identified	0.86	[0.709; 0.949]
Competence → Integrated	0.76	[0.612; 0.840]
Competence → Intrinsic motivation	0.84	[0.717; 0.902]
Relatedness → Amotivation	−0.35	[−0.712; −0.183]
Relatedness → External	−0.17	[−0.513; −0.010]
Relatedness → Introjected	0.02	[−0.116; 0.156]
Relatedness → Identified	0.51	[0.294; 0.983]
Relatedness → Integrated	0.46	[0.294; 0.879]
Relatedness → Intrinsic motivation	0.49	[0.317; 0.920]
*Needs Frustration*		
Autonomy → Amotivation	0.71	[0.623; 0.783]
Autonomy → External	0.66	[0.525; 0.758]
Autonomy → Introjected	0.14	[−0.075; 0.323]
Autonomy → Identified	−0.77	[−0.907; −0.548]
Autonomy → Integrated	−0.70	[−0.835; −0.514]
Autonomy → Intrinsic motivation	−0.79	[−0.899; −0.682]
Competence → Amotivation	0.75	[0.658; 0.832]
Competence → External	0.68	[0.550; 0.777]
Competence → Introjected	0.20	[−0.102; 0.403]
Competence → Identified	−0.75	[−0.944; −0.450]
Competence → Integrated	−0.62	[−0.835; −0.382]
Competence → Intrinsic motivation	−0.69	[−0.874; −0.450]
Relatedness → Amotivation	0.82	[0.736; 0.888]
Relatedness → External	0.67	[0.557; 0.773]
Relatedness → Introjected	0.17	[−0.055; 0.341]
Relatedness → Identified	−0.73	[−0.930; −0.486]
Relatedness → Integrated	−0.54	[−0.784; −0.322]
Relatedness → Intrinsic motivation	−0.63	[−0.834; −0.418]

**Notes.** β = standardized coefficients; CI 95% = Confidence Interval at 95%.

**Table 5:** Multigroup analysis groups.

Models	χ^2^	∆χ^2^	df	∆df	*p*	CFI	∆CFI	SRMR	∆SRMR	RMSEA	∆RMSEA
Swimming—Football											
Configural Invariance	894.318	-	474	-	-	0.925	-	0.058	-	0.039	-
Metric Invariance	934.318	39.647	492	18	0.002	0.922	0.003	0.062	0.004	0.039	0.000
Scalar Invariance	1045.633	150.961	513	39	<0.001	0.916	0.009	0.067	0.009	0.042	0.003
Residual Invariance	1180.794	286.122	537	63	<0.001	0.908	0.017	0.067	0.009	0.045	0.006
13–15—>16 years old											
Configural Invariance	887.316	-	474	-	-	0.925	-	0.055	-	0.038	-
Metric Invariance	936.310	48.994	492	18	<0.001	0.919	0.006	0.056	0.001	0.039	0.001
Scalar Invariance	1045.408	158.092	513	39	<0.001	0.915	0.010	0.061	0.006	0.042	0.004
Residual Invariance	1208.912	321.596	537	63	<0.001	0.898	0.027	0.064	0.009	0.046	0.008
<10 years—≥10 years											
Configural Invariance	886.291	-	474	-	-	0.932	-	0.060	-	0.038	-
Metric Invariance	917.282	30.992	492	18	0.029	0.930	0.002	0.062	0.002	0.038	0.000
Scalar Invariance	945.329	59.039	513	39	0.021	0.929	0.003	0.065	0.005	0.038	0.000
Residual Invariance	1026.146	139.855	537	63	<0.001	0.919	0.013	0.070	0.010	0.039	0.001
Male—Female											
Configural Invariance	876.711	-	474	-	-	0.935	-	0.052	-	0.037	-
Metric Invariance	891.526	14.814	492	18	0.675	0.935	0.000	0.055	0.003	0.038	0.001
Scalar Invariance	948.287	71.576	513	39	0.001	0.930	0.005	0.060	0.008	0.038	0.001
Residual Invariance	141.921	141.921	537	63	<0.001	0.922	0.013	0.062	0.010	0.039	0.002

**Notes**. *p* = level of significance; ∆ = differences among configural and nested models.
